# Supplementary material for: Discovery of New 2-Phenylamino-3-acyl-1,4-naphthoquinones as Inhibitors of Cancer Cells Proliferation: Searching for Intra-Cellular Targets Playing a Role in Cancer Cells Survival
Source: Molecules. 2023 May 24;28(11):4323. doi: 10.3390/molecules28114323 (PMC10254843; doi:10.3390/molecules28114323)
Supplement: Supplementary file 1 [file molecules-28-04323-s001.zip › molecules-2396822-supplementary.pdf]

Supplementary Materials

Discovery of New 2-Phenylamino-3-acyl-1,4-naphthoquinones as Inhibitors of Cancer Cells Proliferation: Searching for Intra-Cellular Targets Playing a Role in Cancer Cells Survival

Julio Benites, Jaime A. Valderrama, Álvaro Contreras, Cinthya Enríquez, Ricardo Pino-Rios, Osvaldo Yáñez and Pedro Buc Calderon

2-(Phenylamino)-3-hexanoylnaphthalene-1,4-dione **1**. ^{1}H−NMR and ^{13}C−NMR

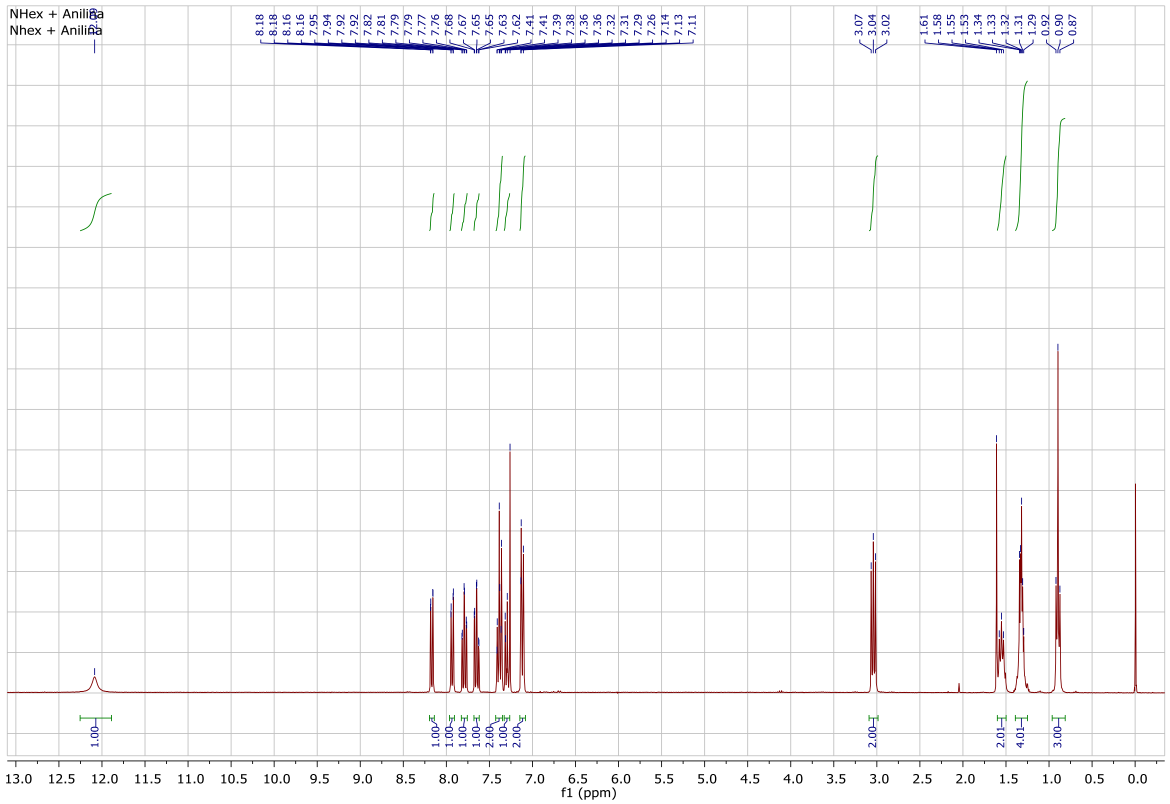

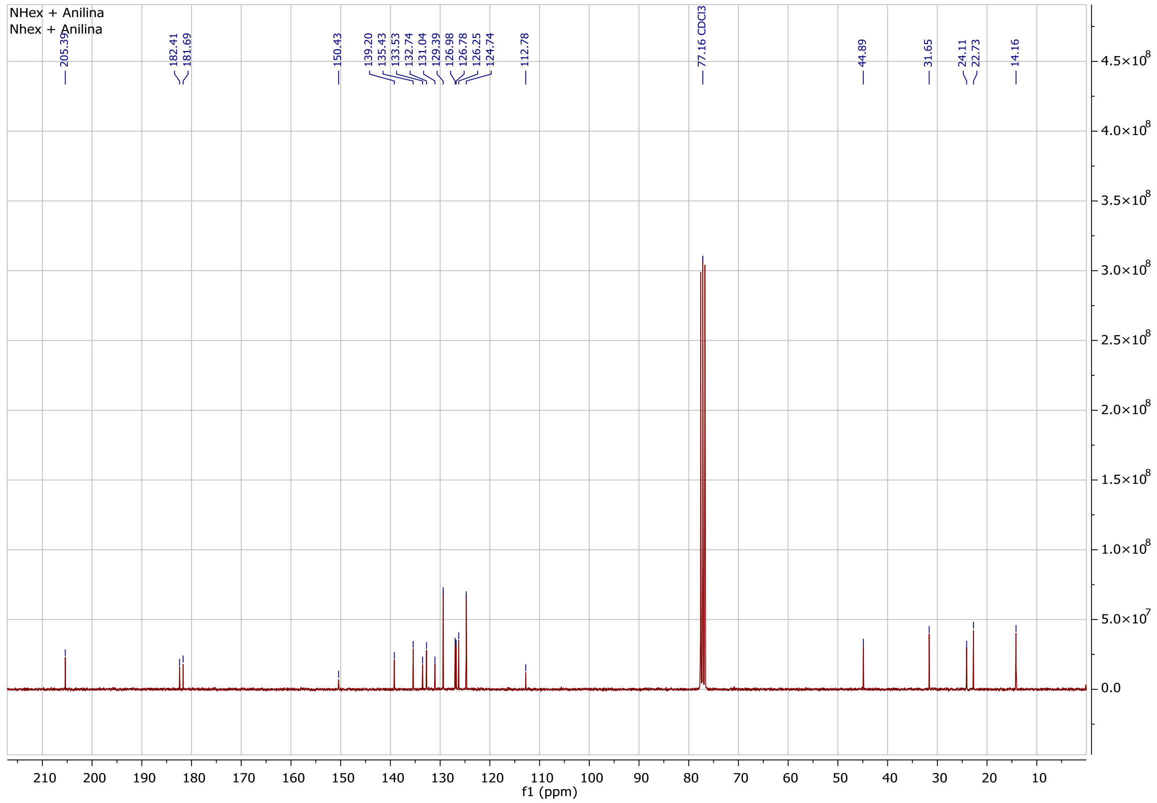

2-(Phenylamino)-3-decanoylnaphthalene-1,4-dione **2.** ^{1}H-NMR and ^{13}C NMR

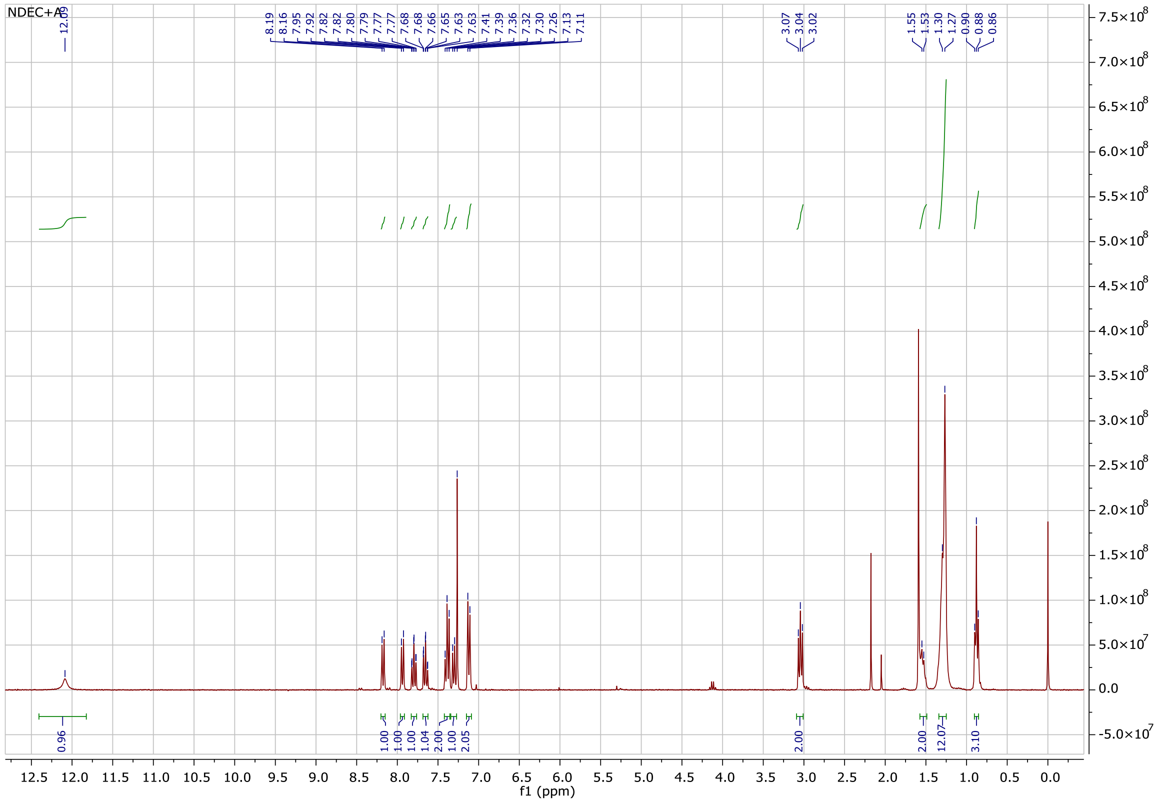

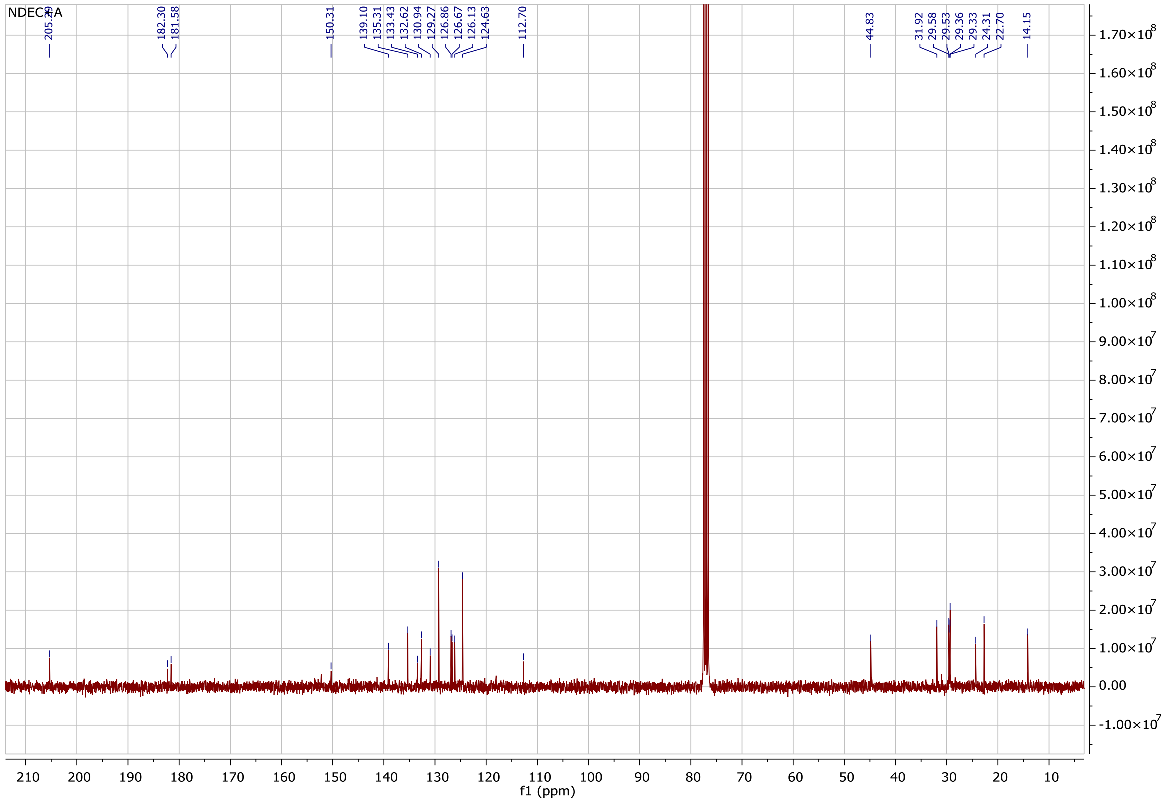

2-(Phenylamino)-3-dodecanoylnaphthalene-1,4-dione **3.** ^{1}H-NMR and ^{13}C-NMR

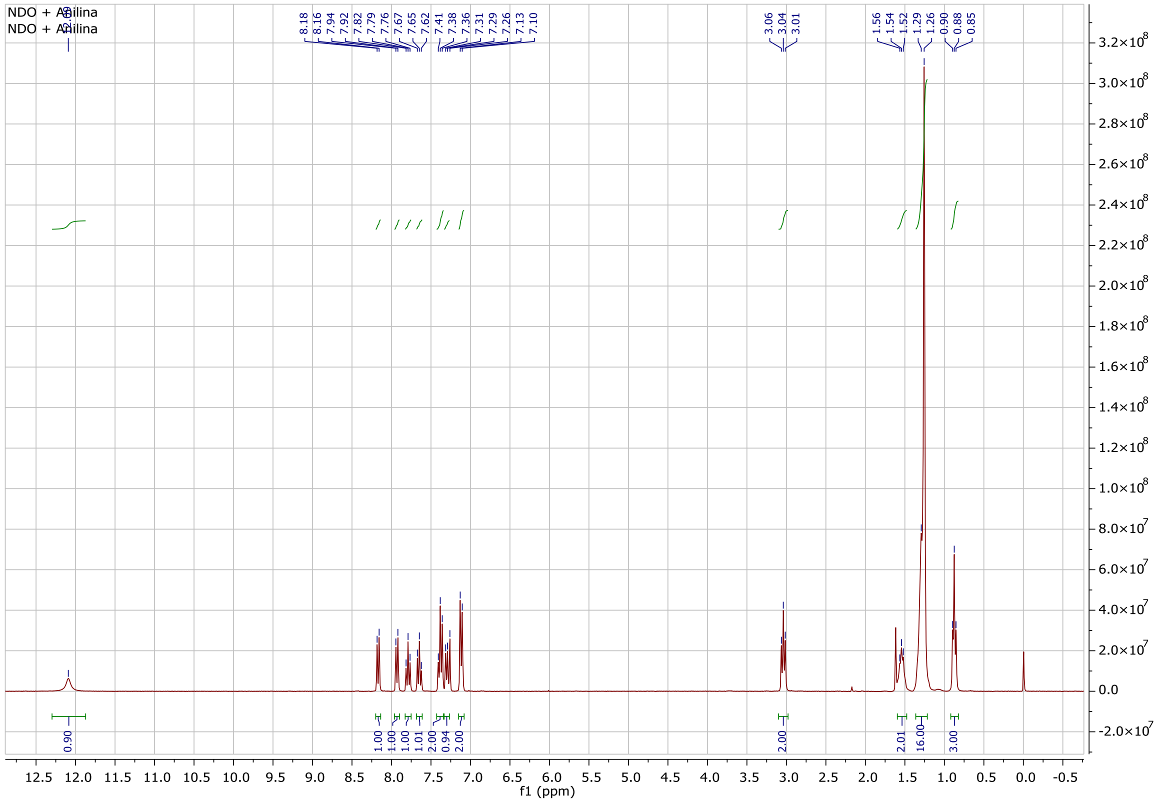

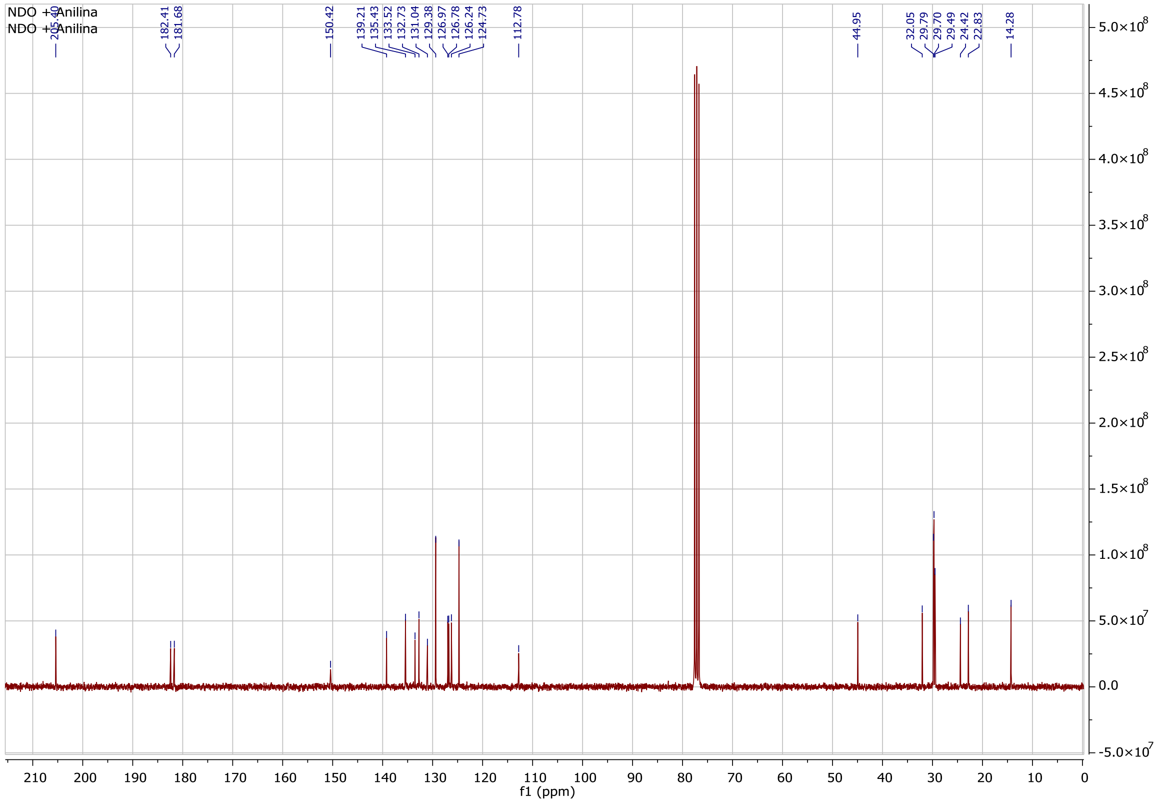

2-(Phenylamino)-3-benzoylnaphthalene-1,4-dione **4.** ^{1}H-NMR and ^{13}C-NMR

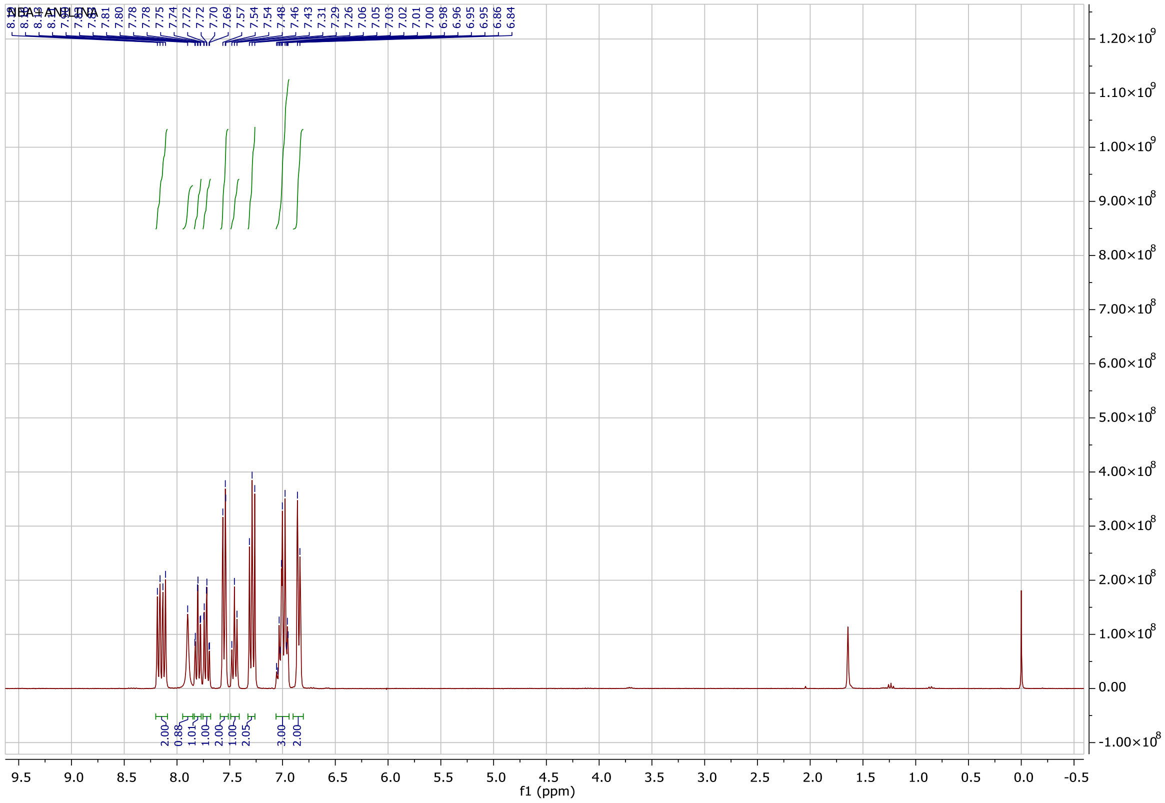

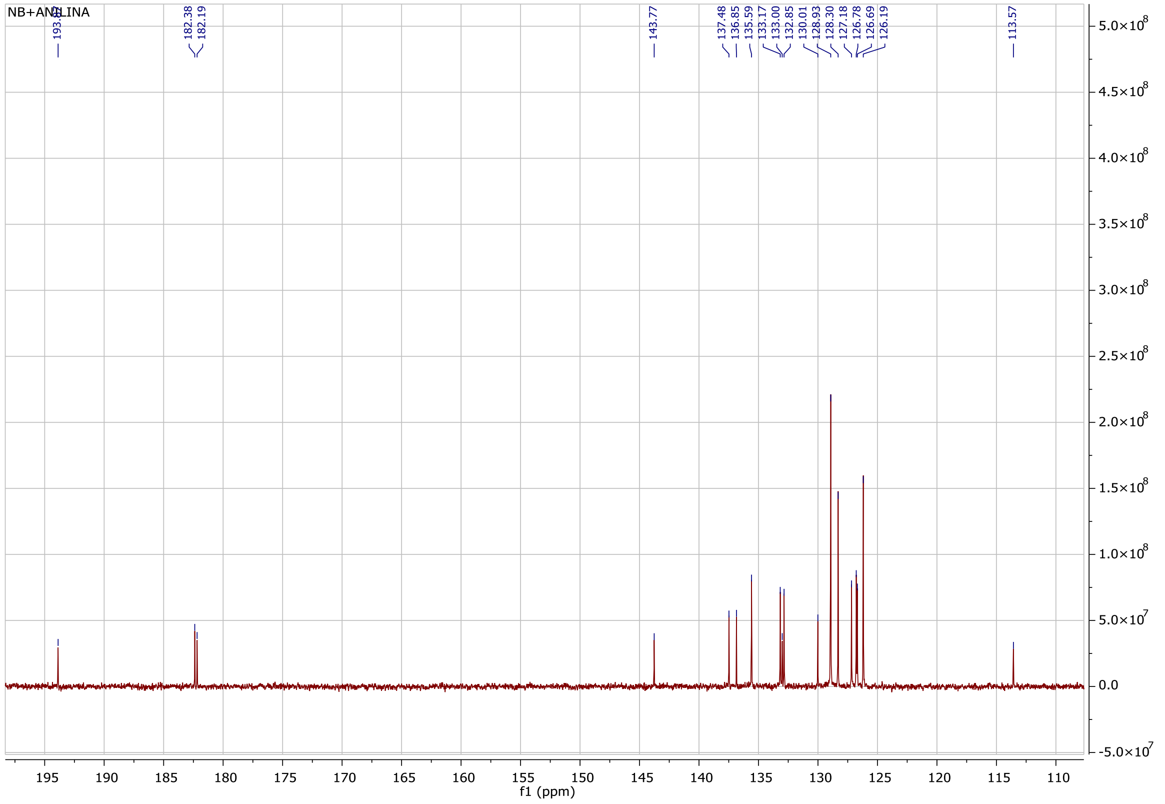

2-(phenylamino)-3-(3-methoxybenzoyl)naphthalene-1,4-dione **5.** ^{1}H-NMR and ^{13}C-NMR

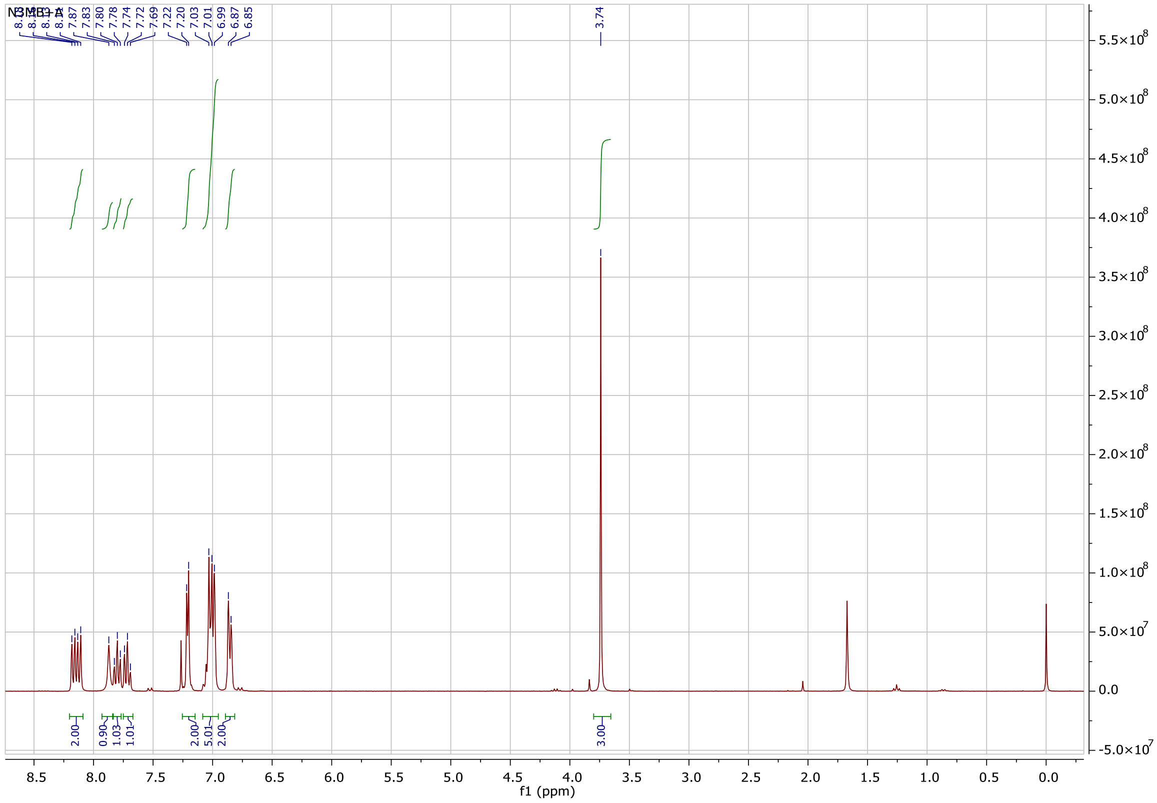

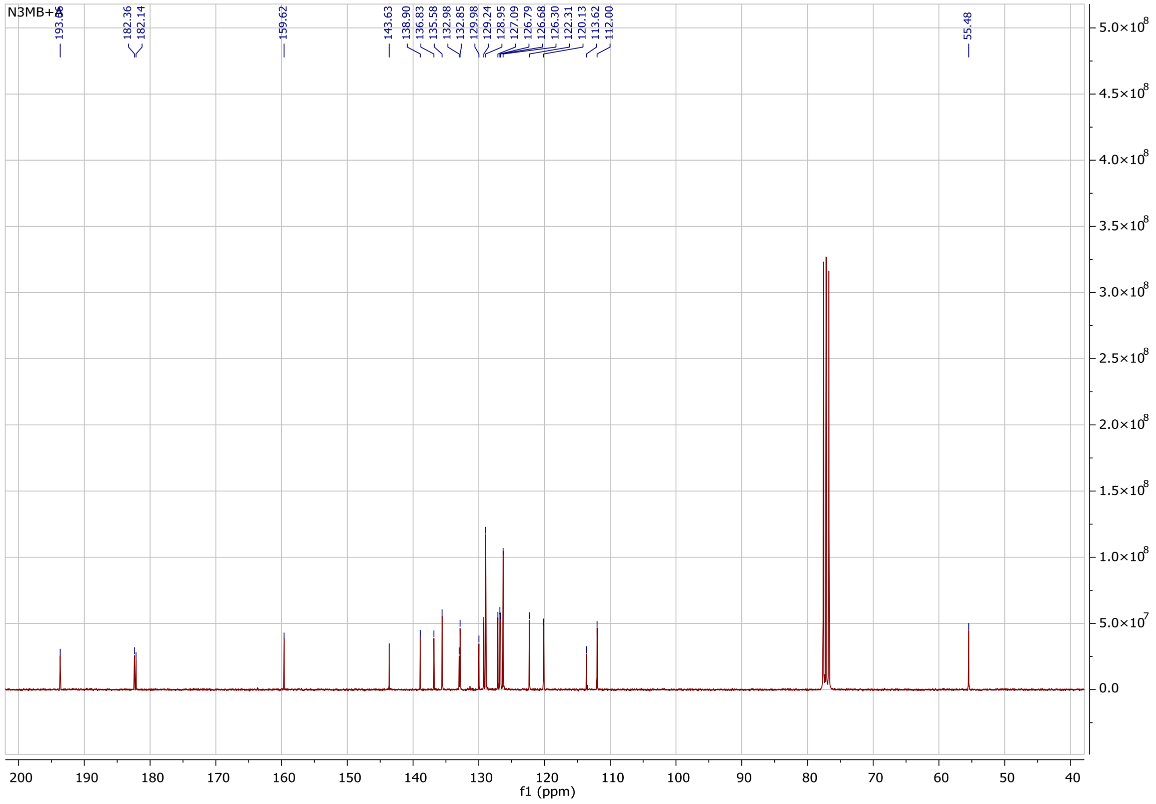

2-(Phenylamino)-3-(4-methoxybenzoyl)naphthalene-1,4-dione **6.** ^{1}H-NMR and ^{13}C-RMN

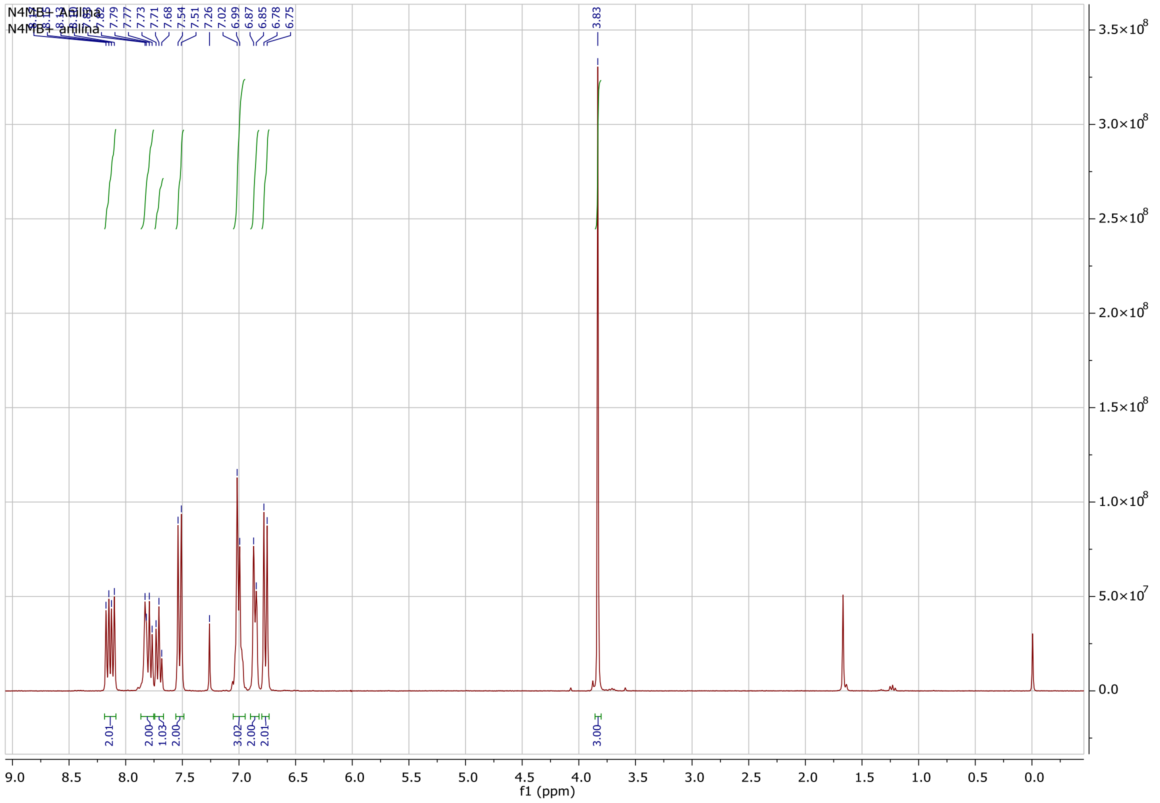

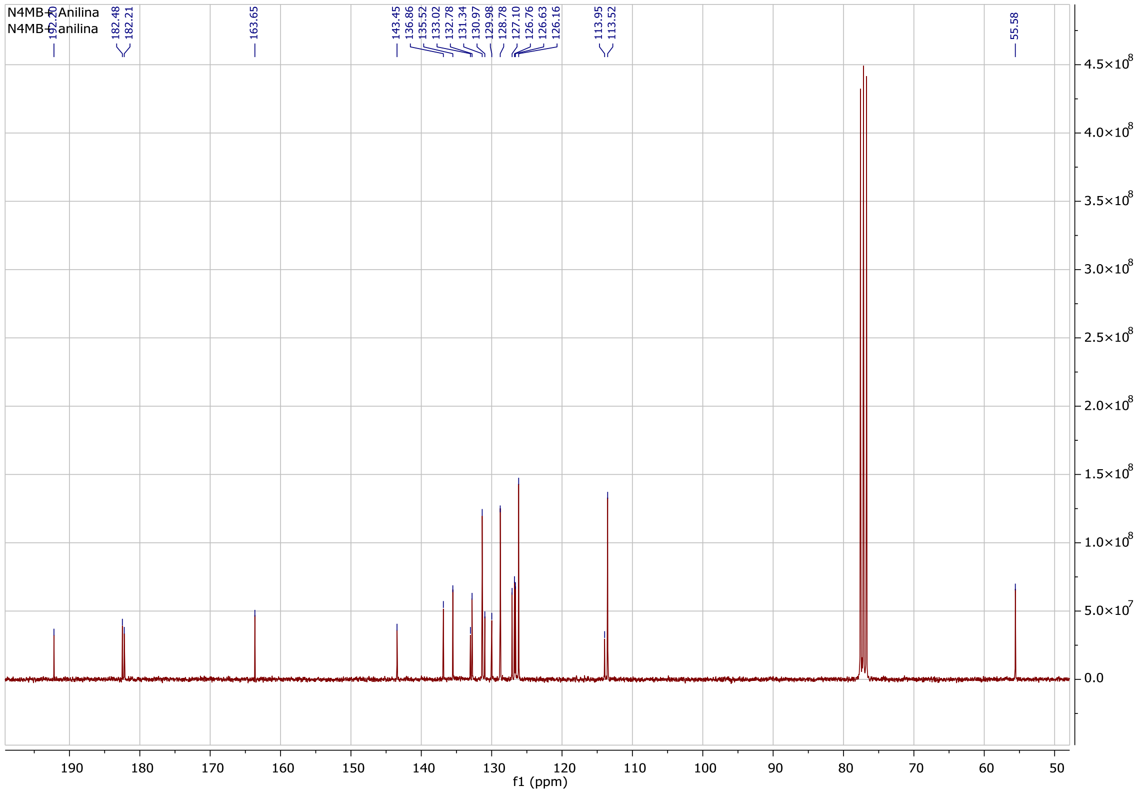

2-(Phenylamino)-3-(3,4-dimethoxybenzoyl)naphthalene-1,4-dione **7.** ^{1}H-NMR and ^{13}C-NMR

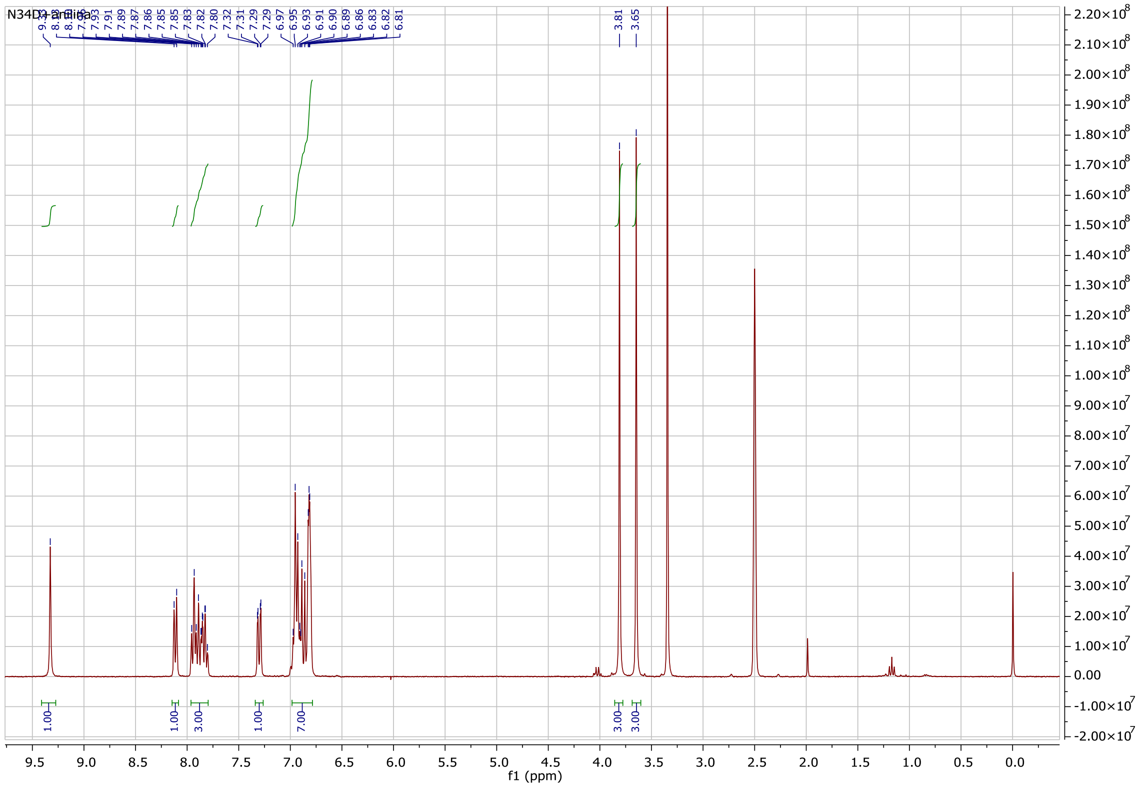

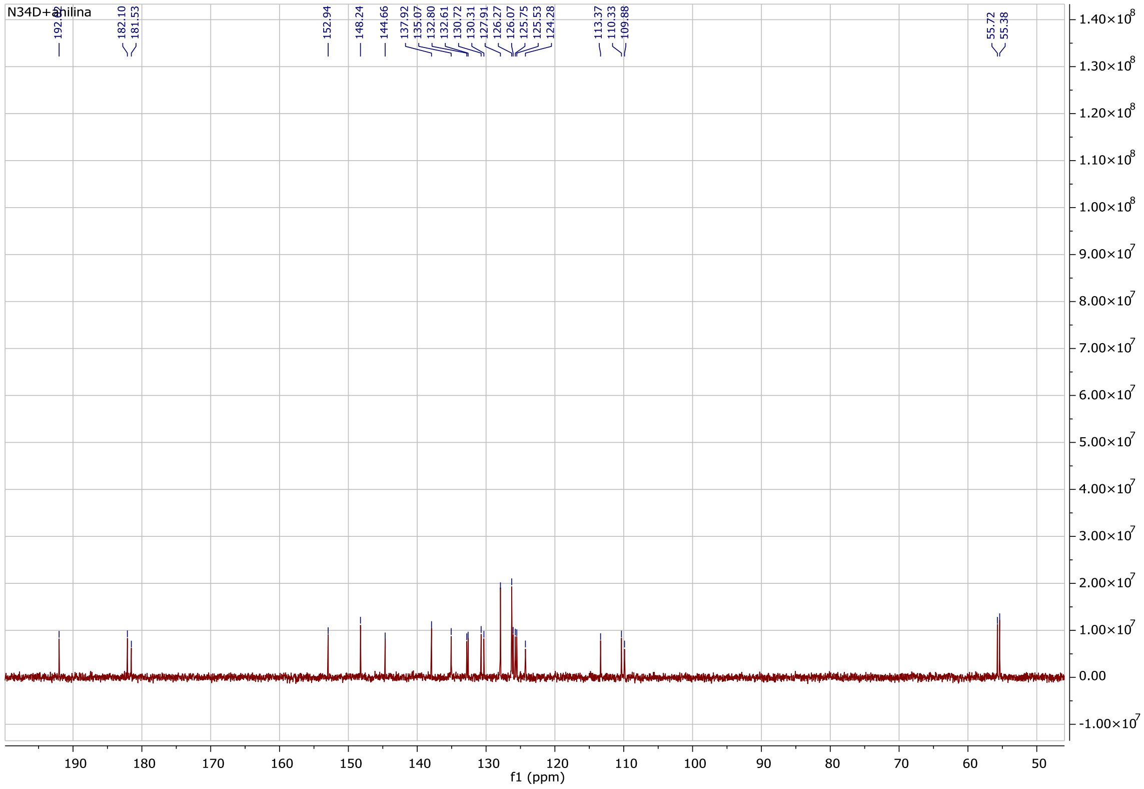

2-(Phenylamino)-3-(3,4,5-trimethoxybenzoyl)naphthalene-1,4-dione **8**. ^{1}H-NMR and ^{13}C-NMR

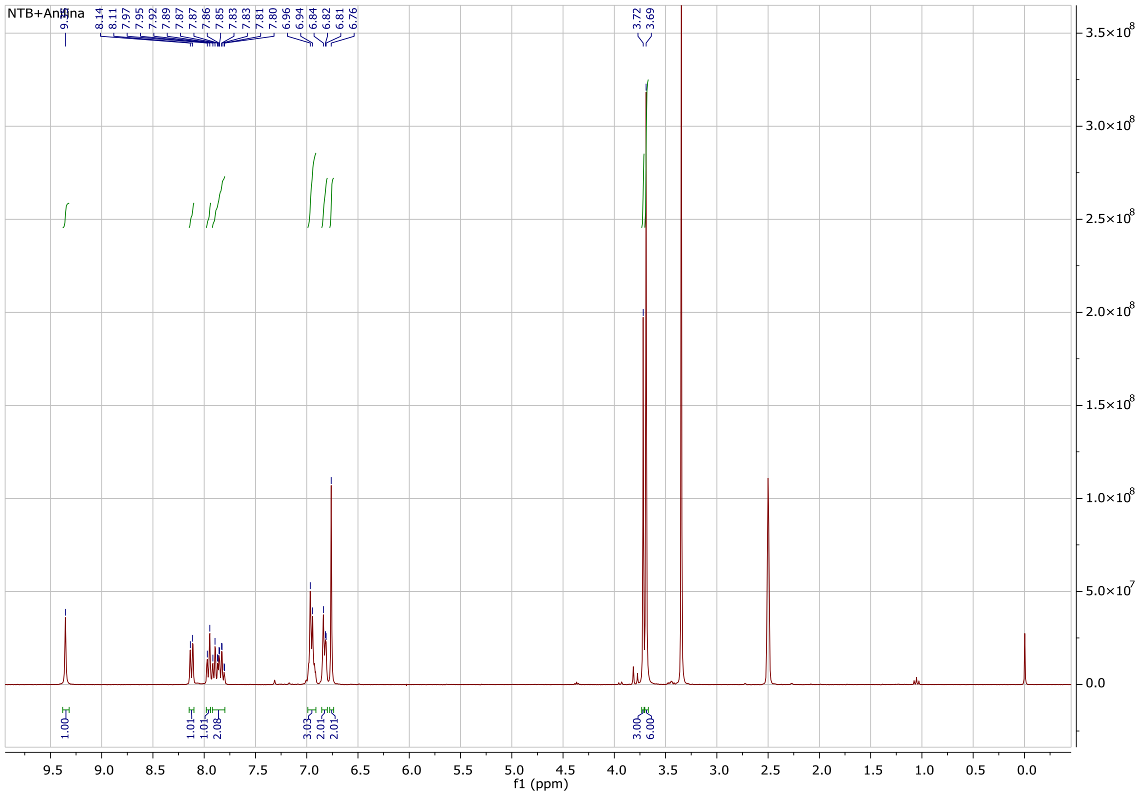

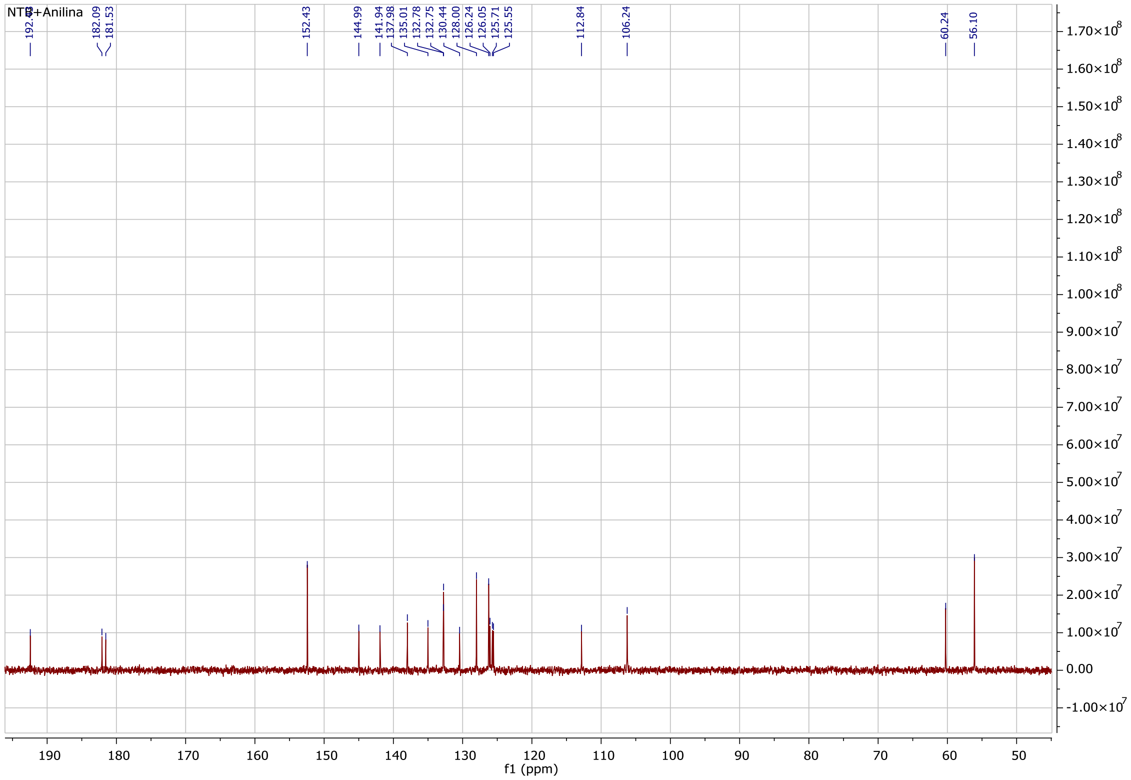

2-(Phenylamino)-3-(4-hydroxy-3-methoxybenzoyl)naphthalene-1,4-dione **9.** ^{1}H-NMR and ^{13}C-NMR

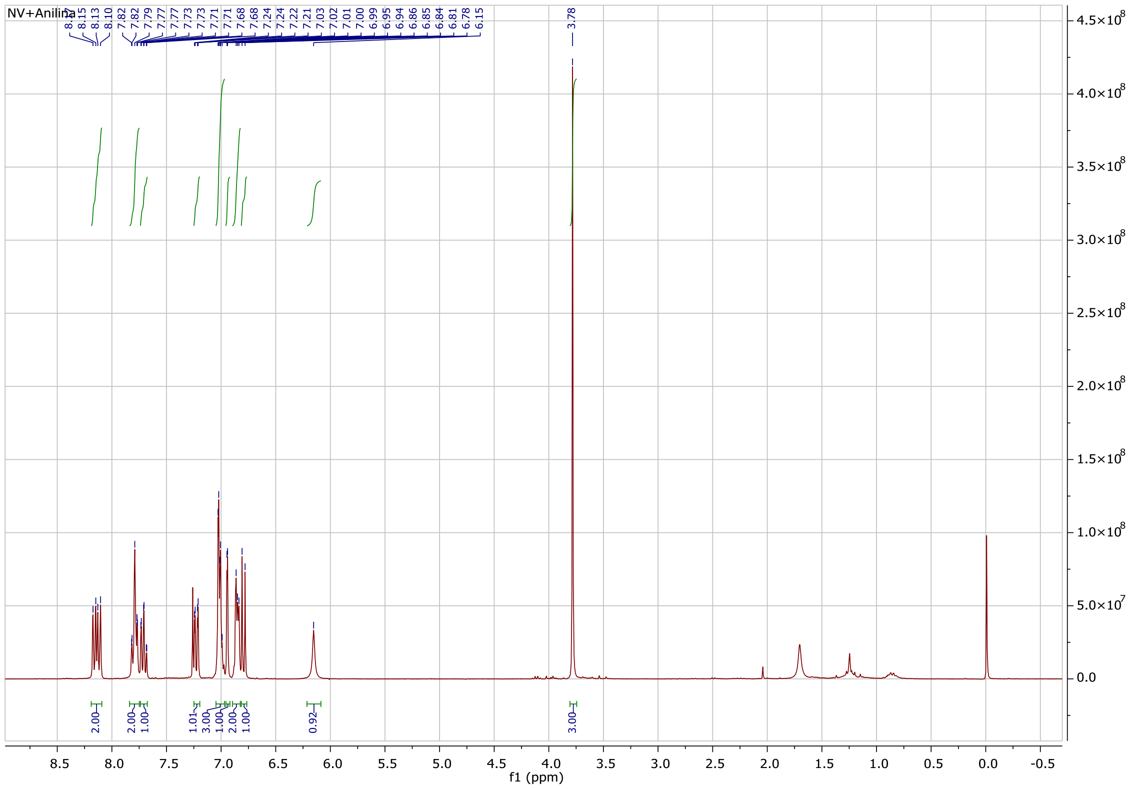

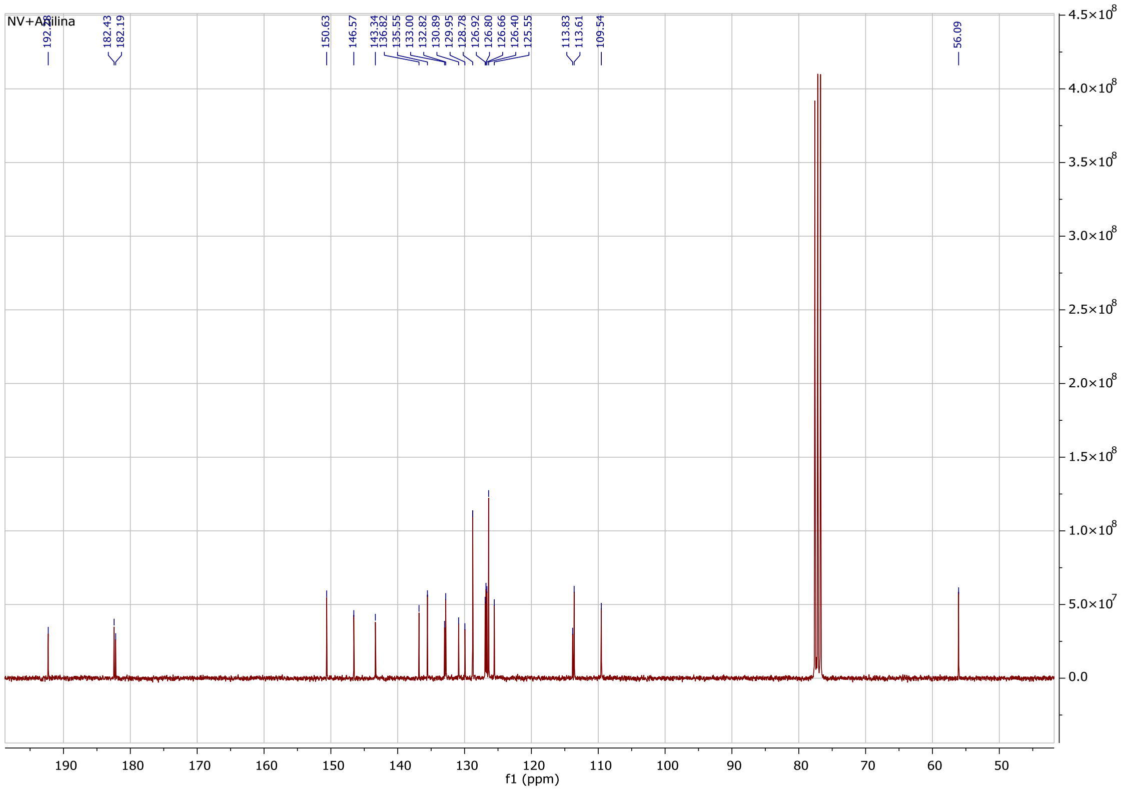

2-(Phenylamino)-3-(thiophene-3-carbonyl)naphthalene-1,4-dione **13.** ^{1}H-NMR and ^{13}C-NMR

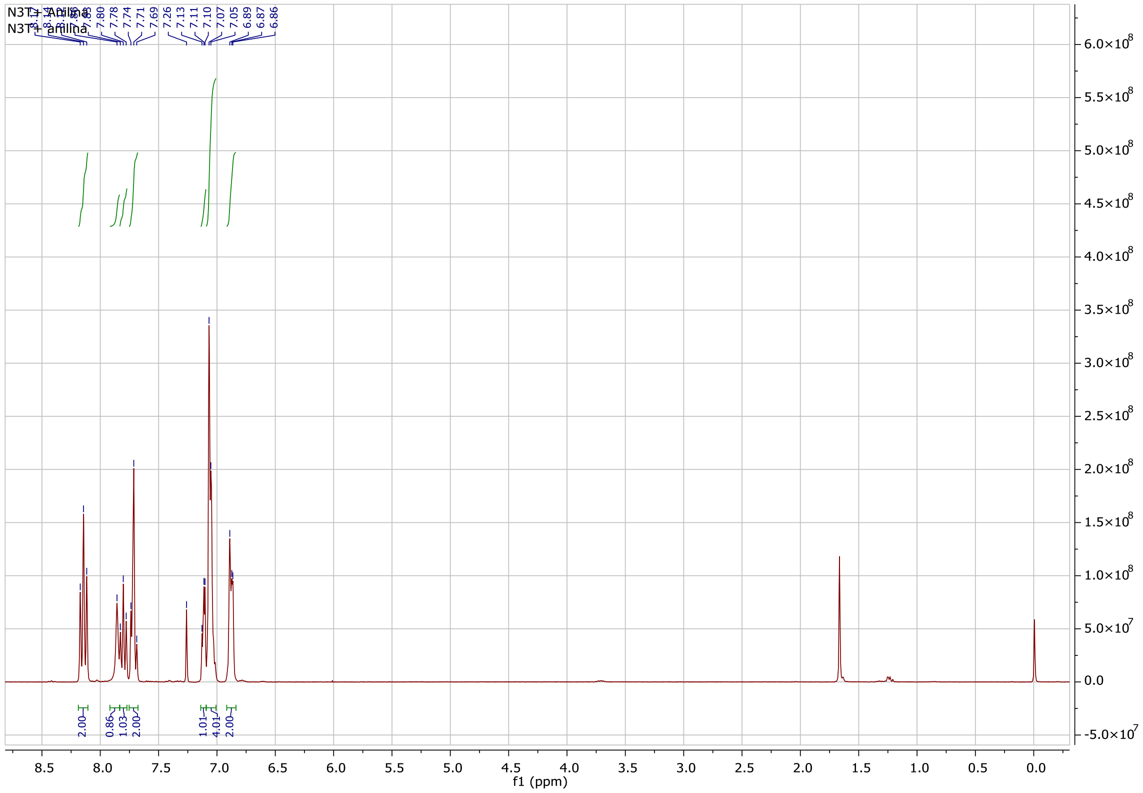

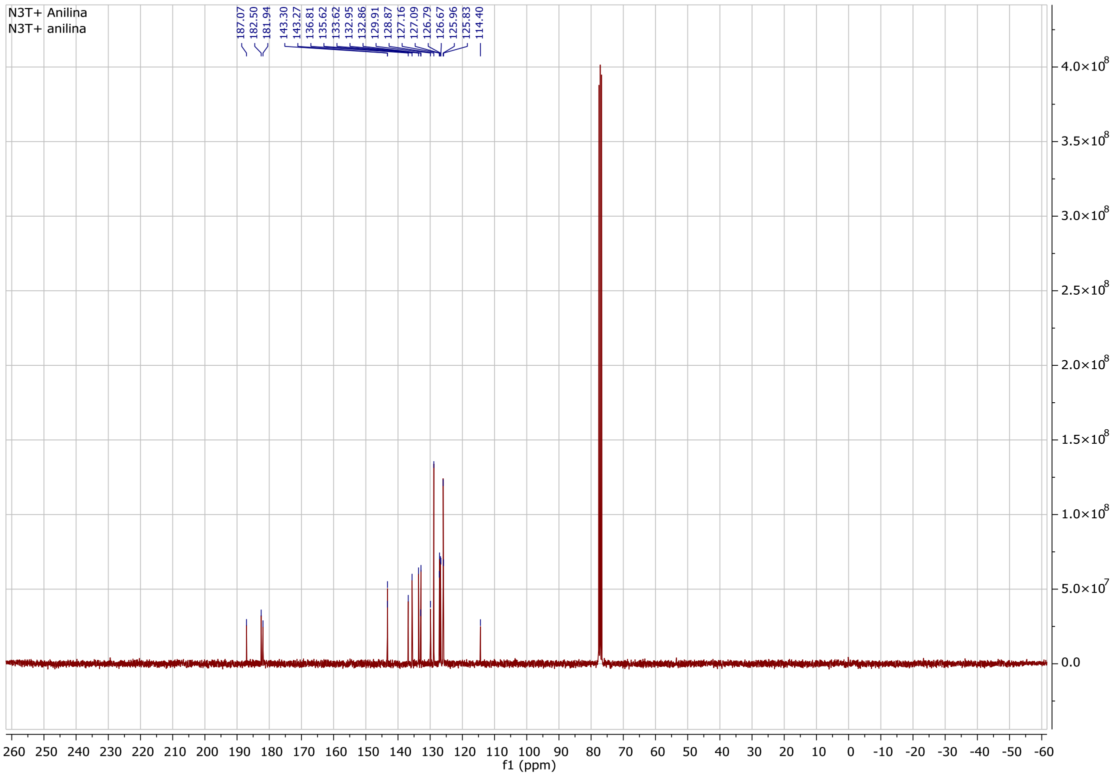

2-(Phenylamino)-3-(1H-pyrrole-2-carbonyl)naphthalene-1,4-dione **14.** ^{1}H-NMR and ^{13}C-NMR

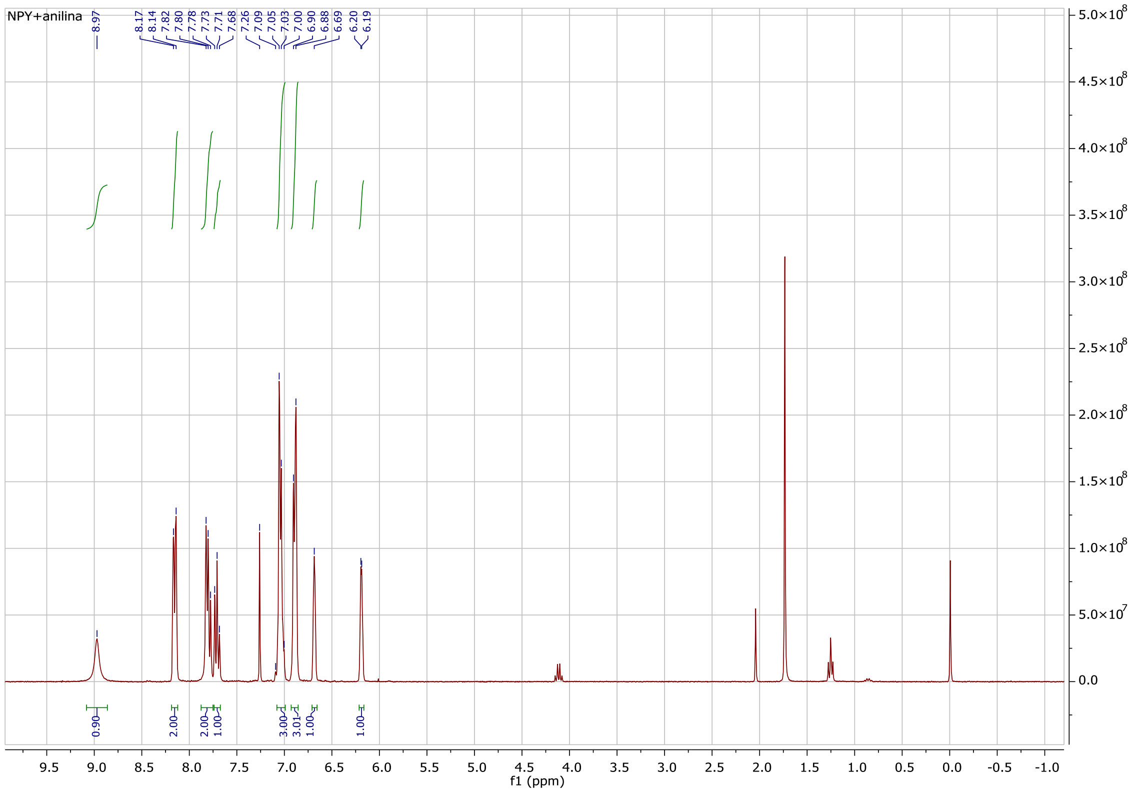

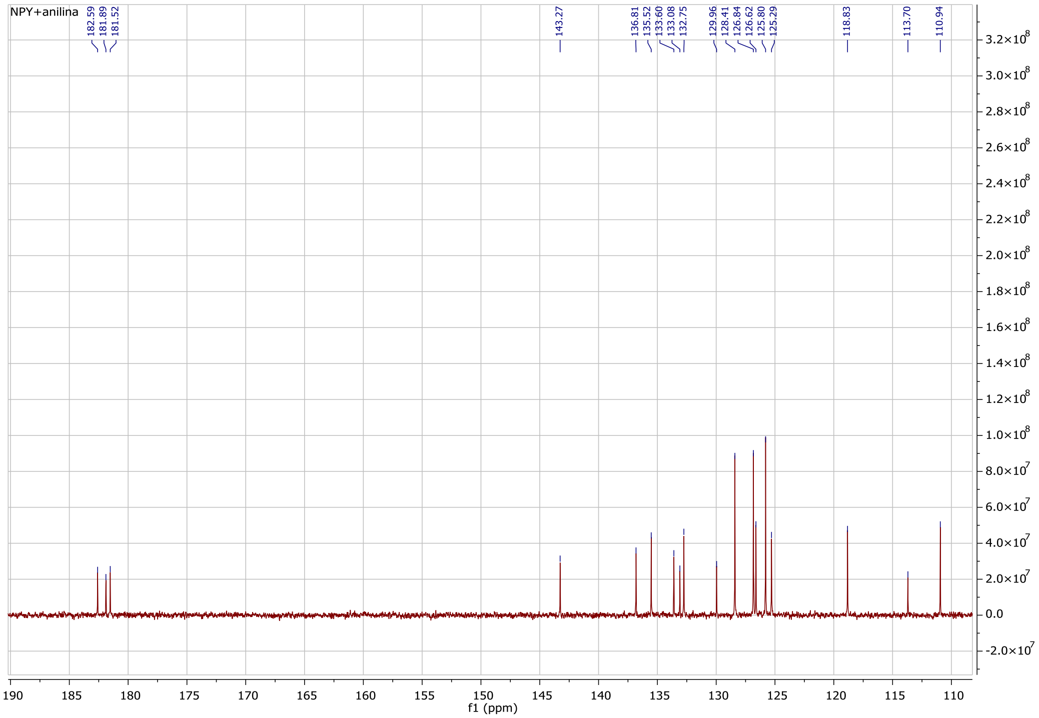

Figure S1. ^{1}H- and ^{13}C- NMR spectra of new synthesize compounds

Comp 4- MCF-7

Nonlin fit	A
	MCF-7 Comp4
	Y
log(inhibitor) vs. normalized response -- Variable slope	
Best-fit values	
LogIC50	0.7178
HillSlope	-1.925
IC50	5.222
Std. Error	
LogIC50	0.05491
HillSlope	0.2801
95% Confidence Intervals	
LogIC50	0.6076 to 0.8281
HillSlope	-2.487 to -1.362
IC50	4.051 to 6.732
Goodness of Fit	
Degrees of Freedom	52
R square	0.9405
Absolute Sum of Squares	5151
Sy.x	9.953
Number of points	
Analyzed	54

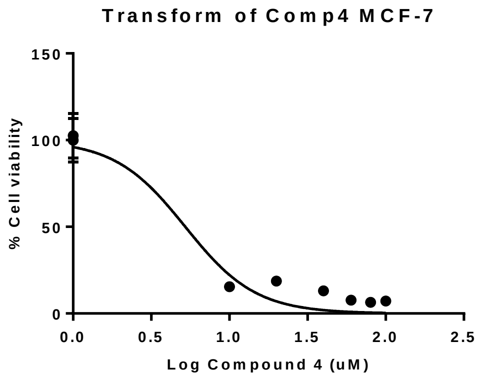

Comp 4-T24

Nonlin fit	A
	Data Set-A
	Y
log(inhibitor) vs. normalized response -- Variable slope	
Best-fit values	
LogIC50	1.221
HillSlope	-0.9122
IC50	16.64
Std. Error	
LogIC50	0.02232
HillSlope	0.04252
95% Confidence Intervals	
LogIC50	1.177 to 1.266
HillSlope	-0.9972 to -0.8273
IC50	15.02 to 18.44
Goodness of Fit	
Degrees of Freedom	66
R square	0.9629
Absolute Sum of Squares	2461
Sy.x	6.107
Number of points	
Analyzed	68

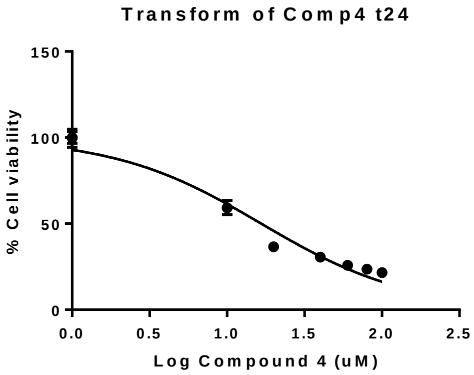

Comp 4 – DU 145

Nonlin fit	A Data Set-A Y
log(inhibitor) vs. normalized response -- Variable slope	
Best-fit values	
LogIC50	-0.2250
HillSlope	-0.4817
IC50	0.5957
Std. Error	
LogIC50	0.1067
HillSlope	0.04291
95% Confidence Intervals	
LogIC50	-0.4390 to -0.01087
HillSlope	-0.5678 to -0.3955
IC50	0.3639 to 0.9753
Goodness of Fit	
Degrees of Freedom	53
R square	0.7308
Absolute Sum of Squares	3165
Sy.x	7.727
Number of points	
Analyzed	55

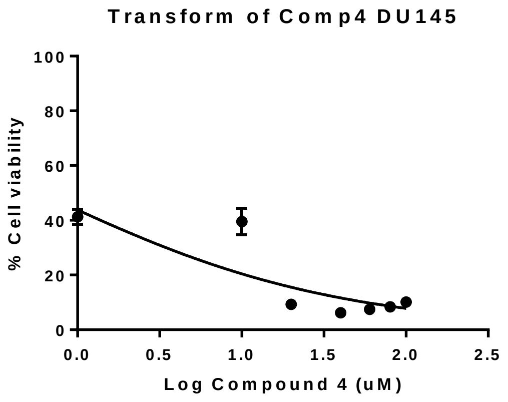

Comp 4 – HEK 293

Nonlin fit	A Data Set-A Y
log(inhibitor) vs. normalized response -- Variable slope	
Best-fit values	
LogIC50	1.639
HillSlope	-1.236
IC50	43.53
Std. Error	
LogIC50	0.01642
HillSlope	0.07983
95% Confidence Intervals	
LogIC50	1.606 to 1.672
HillSlope	-1.397 to -1.076
IC50	40.35 to 46.97
Goodness of Fit	
Degrees of Freedom	54
R square	0.9527
Absolute Sum of Squares	2200
Sy.x	6.383
Number of points	
Analyzed	56

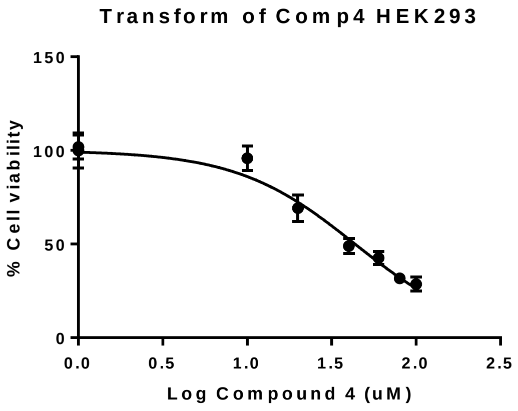

Figure S2. Graphing dose-response curves of compound **4**

Comp 11- MCF-7

Nonlin fit	A
	Data Set-A
	Y
log(inhibitor) vs. normalized response -- Variable slope	
Best-fit values	
LogIC50	0.7079
HillSlope	-2.012
IC50	5.104
Std. Error	
LogIC50	0.02923
HillSlope	0.1535
95% Confidence Intervals	
LogIC50	0.6494 to 0.7663
HillSlope	-2.319 to -1.705
IC50	4.461 to 5.839
Goodness of Fit	
Degrees of Freedom	60
R square	0.9833
Absolute Sum of Squares	1516
Sy.x	5.027
Number of points	
Analyzed	62

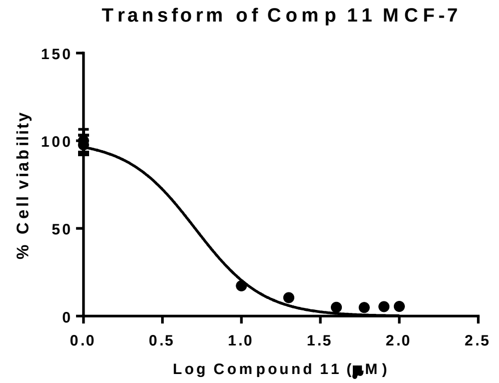

Comp 11 – T24

Nonlin fit	A
	Data Set-A
	Y
log(inhibitor) vs. normalized response -- Variable slope	
Best-fit values	
LogIC50	1.037
HillSlope	-1.331
IC50	10.90
Std. Error	
LogIC50	0.02204
HillSlope	0.07164
95% Confidence Intervals	
LogIC50	0.9931 to 1.082
HillSlope	-1.475 to -1.187
IC50	9.842 to 12.06
Goodness of Fit	
Degrees of Freedom	54
R square	0.9795
Absolute Sum of Squares	1509
Sy.x	5.286
Number of points	
Analyzed	56

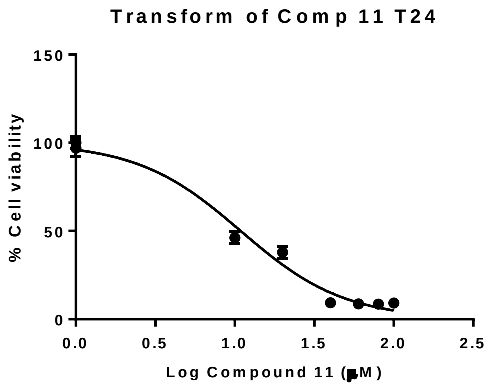

Comp 11 – DU 145

Nonlin fit	A
	Data Set-A
	Y
log(inhibitor) vs. normalized response -- Variable slope	
Best-fit values	
LogIC50	0.6072
HillSlope	-2.306
IC50	4.048
Std. Error	
LogIC50	0.03873
HillSlope	0.1977
95% Confidence Intervals	
LogIC50	0.5297 to 0.6847
HillSlope	-2.701 to -1.910
IC50	3.386 to 4.838
Goodness of Fit	
Degrees of Freedom	59
R square	0.9772
Absolute Sum of Squares	1821
Sy.x	5.555
Number of points	
Analyzed	61

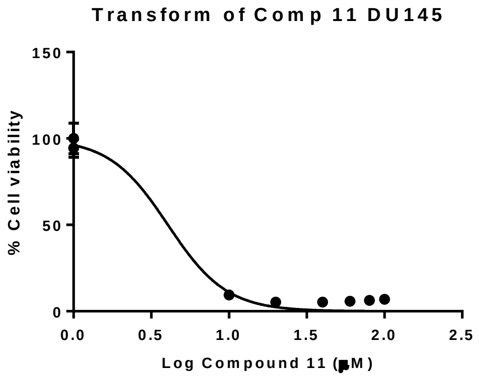

Comp 11 – HEK 293

Nonlin fit	A
	Data Set-A
	Y
log(inhibitor) vs. normalized response -- Variable slope	
Best-fit values	
LogIC50	2.226
HillSlope	-1.036
IC50	168.4
Std. Error	
LogIC50	0.04462
HillSlope	0.1066
95% Confidence Intervals	
LogIC50	2.136 to 2.316
HillSlope	-1.250 to -0.8214
IC50	136.9 to 207.0
Goodness of Fit	
Degrees of Freedom	49
R square	0.8628
Absolute Sum of Squares	1374
Sy.x	5.296
Number of points	
Analyzed	51

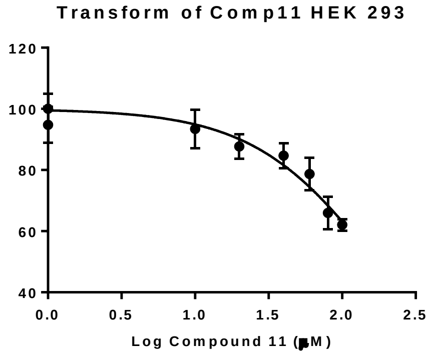

Figure S3. Graphing dose-response curves of compound **11**

Table S1. Quantitative real-time (qPCR) Primer Sequences.

Gene name	Gene symbol	Entrez Gene ID	Forward (5' → 3')	Reverse (5' → 3')	Amplicon Size (pb)
**Apoptosis**					
Apoptosis regulator (BCL2), transcript variant alpha	*BCL2*	NM_000633.2	ATGTGTGTGGAGAGCGTCAA	GAGACAGCCAGGAGAAATCAA	181
**PI3 Kinases & Phosphatases**					
Mechanistic target of rapamycin kinase	*MTOR*	NM_004958.3	TCCGAGAGATGAGTCAAGAGG	CACCTTCCACTCCTATGAGGC	141
**Drug metabolism/Oxidative estres**					
Glutathione-disulfide reductase	*GSR*	NM_000637.4	CACTTGCGTGAATGTTGGATG	TGGGATCACTCGTGAAGGCT	242
**Cell Cycle**					
Cell division cycle 25A	*CDC25A*	NM_001789.2	TGGGCCATTGGACAGTAAAG	TCCCAACAGCTTCTGAGGTA	76
Tumor protein p53	*TP53*	NM_000546.5	ACAGCTTTGAGGTGCGTGTTT	CCCTTTCTTGCGGAGATTCTCT	77
**Hippo Signaling pathway**					
Cellular communication network factor 2	*CCN2*	NM_001901.2	GTACCAGCAGAAAGGTTAGTATCA	GGTCAGTGAGCACGCTAAA	104
**Histone Deacetylases**					
Histone deacetylase 4	*HDAC4*	NM_006037.3	AGCGTCCGTTGGATGTCAC	CCTTCTCGTGCCACAAGTCT	169
**Inflammation**					
Tumor necrosis factor	*TNF*	NM_000594.3	AGAACTCACTGGGGCCTACA	GCTCCGTGTCTCAAGGAAGT	177
**Housekeeping**					
Beta-2-microglobulin	*B2M*	NM_004048.2	ATGAGTATGCCTGCCGTGTGA	GGCATCTTCAAACCTCCATG	97

Table S2: Full reports of SwissADME and pkCSM parameters

SwissADME PARAMETERS		
**COMPOUND**	**4**	**11**
Structure	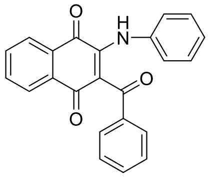	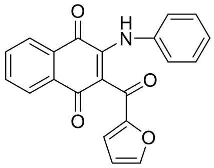
PHYSICOCHEMICAL PROPERTIES		
Formula	$C_{23}H_{15}NO_3$	$C_{21}H_{13}NO_4$
Molecular weight	353.37 g/mol	343.33 g/mol
Num. heavy atoms	27	26
Num. arom. heavy atoms	18	17
Fraction Csp^3	0.00	0.00
Num. rotatable bonds	4	4
Num. H-bond acceptors	3	4
Num. H-bond donors	1	1
Molar Refractivity	102.79	95.05
TPSA	63.24 Å²	76.38 Å²
LIPOPHILICITY		
Log $P_{o/w}$ (iLOGP)	2.56	2.19
Log $P_{o/w}$ (XLOGP3)	5.49	4.70
Log $P_{o/w}$ (WLOGP)	4.12	3.72
Log $P_{o/w}$ (MLOGP)	2.14	0.91
Log $P_{o/w}$ (SILICOS-IT)	4.49	3.87
Consensus Log $P_{o/w}$	3.76	3.08
WATER SOLUBILITY		
Log *S* (ESOL)	-5.72	-5.15
Solubility	6.75e-04 mg/mL; 1.91e-06 mol/L	2.43e-03 mg/mL; 7.09e-06 mol/L

Class	Moderately soluble	Moderately soluble
Log *S* (Ali)	-6.58	-6.03
Solubility	9.38e-05 mg/ml; 2.65e-07 mol/L	3.19e-04 mg/ml; 9.28e-07 mol/L
Class	Poorly soluble	Poorly soluble
Log *S* (SILICOS-IT)	-8.25	-7.47
Solubility	2.01e-06 mg/mL; 5.68e-09 mol/L	1.17e-05 mg/mL; 3.41e-08 mol/L
Class	Poorly soluble	Poorly soluble
PHARMACOKINETICS		
GI absorption	High	High
BBB permeant	Yes	Yes
P-gp substrate	No	No
CYP1A2 inhibitor	Yes	Yes
CYP2C19 inhibitor	Yes	Yes
CYP2C9 inhibitor	Yes	Yes
CYP2D6 inhibitor	No	No
CYP3A4 inhibitor	Yes	Yes
Log *K*p (skin permeation)	-4.56 cm/s	-5.06 cm/s
DRUGLIKENESS		
Lipinski	Yes; 0 violation	Yes; 0 violation
Ghose	Yes	Yes
Veber	Yes	Yes
Egan	Yes	Yes
Muegge	No; 1 violation: XLOGP3>5	Yes
Bioavailability Score	0.55	0.55
MEDICINAL CHEMISTRY		
PAINS	1 alert: quinone_A	1 alert: quinone_A
Brenk	1 alert: michael_acceptor_4	1 alert: michael_acceptor_4
Leadlikeness	No; 2 violations: MW>350, XLOGP3>3.5	No; 1 violation: XLOGP3>3.5

Synthetic accessibility	3.13	3.34
**pkCSM PARAMETERS**		
ABSORPTION		
Water solubility (log mol/L)	-4.677	-4.035
Caco2 permeability (log Papp in 10-6 cm/s)	0.483	0.939
Intestinal absorption (human) (%Absorbed)	94.39	94.324
Skin Permeability (log Kp)	-2.774	-2.798
P-glycoprotein substrate	Yes	Yes
P-glycoprotein I inhibitor	Yes	Yes
P-glycoprotein II inhibitor	Yes	Yes
DISTRIBUTION		
VDss (human) (log L/Kg)	-0.105	0.013
Fraction unbound (human) (Fu)	0.016	0.06
BBB permeability (log BB)	0.059	-0.027
CNS permeability (log PS)	-1.738	-1.864
METABOLISM		
CYP2D6 substrate	No	No
CYP3A4 substrate	Yes	Yes
CYP1A2 inhibitor	Yes	Yes
CYP2C19 inhibitor	Yes	Yes
CYP2C9 inhibitor	Yes	Yes
CYP2D6 inhibitor	No	No
CYP3A4 inhibitor	Yes	Yes
EXCRETION		
Total Clearance (log mL/min/Kg)	0.138	0.138
Renal OCT2 substrate	No	No

TOXICITY		
AMES toxicity	Yes	No
Max. tolerated dose (human) (log mg/kg/day)	0.49	0.301
hERG I inhibitor	No	No
hERG II inhibitor	Yes	Yes
Oral Rat Acute Toxicity (LD50) (mol/kg)	2.704	2.779
Oral Rat Chronic Toxicity (LOAEL) (log mg/kg_bw/day)	1.762	1.44
Hepatotoxicity	Yes	No
Skin Sensitization	No	No
T. Pyriformis toxicity (log ug/L)	0.404	0.63
Minnow toxicity (log mM)	-0.021	0.743
